# Elements of dissemination in cryptococcosis

**DOI:** 10.1128/mbio.02155-23

**Published:** 2024-10-29

**Authors:** Joseph M. Bednarek, Jessica C. S. Brown

**Affiliations:** 1 School of Biological Sciences, University of Utah, Salt Lake City, Utah, USA; Instituto Carlos Chagas, Curitiba, Brazil

**Keywords:** medical mycology, dissemination, *Cryptococcus neoformans*, cryptococcosis, fungal meningitis

## Abstract

As healthcare improves and our ability to support patients with compromised immune systems increases, such patients become more vulnerable to microbes in the environment. These include fungal pathogens such as *Cryptococcus neoformans*, the primary cause of fungal meningitis and a top priority pathogen on the World Health Organization fungal pathogen list. Like many other environmental pathogens, *C. neoformans* must adapt to and thrive in diverse environments in order to cause disease: (i) the environmental niche, (ii) the lungs following inhalation of infectious particles, (iii) the bloodstream and/or lymphatic system during dissemination, and (iv) the central nervous system (CNS), where it causes a deadly cryptococcal meningoencephalitis. Because CNS infection is the driver of mortality and the presenting illness, understanding the dissemination process from both host and fungal perspectives is important for treating these infections. In this review, we discuss the different stages of dissemination, how fungal cells interact with host cells during disease, and the ability to adapt to different environments within hosts.

## INTRODUCTION

*Cryptococcus neoformans* is a fungal pathogen with global distribution. This saprophytic fungus is commonly found in soil and arboreal ecosystems, where it sources nutrients from decaying organisms (1). *C. neoformans* is opportunistic, primarily infecting immunocompromised patients. There is no evidence that it can spread between people, so human infection is unlikely to exert selective pressure on *C. neoformans*. Instead, many of the virulence mechanisms it employs are thought to have originated from evolutionary pressures in the environment (2). For example, it exhibits a unique capacity for phagocytic deterrence and survival. This trait is hypothesized to have evolved from interactions with predatory amoebae that coexist with the fungi, as the phagocytic mechanisms used by environmental amoebae are remarkably like those of immune cells (2, 3).

Infections with *C. neoformans* begin in the lungs after the inhalation of fungal particles from the environment. Whether those particles are spores or yeasts remains unclear, but the two forms are both virulent in a murine model of cryptococcosis (4–6). Spores, however, are the more likely source of infection due to their inherent stress resistance and capacity for airborne distribution (6, 7). Once in the lung, the spore germinates to yeast or the yeast cell replicates. The fungal cells are either cleared or persist in a state of latency. In the event of immune suppression, like the onset of AIDS, the latent fungi can escape the lungs and disseminate to the brain. Fungal proliferation in the brain results in meningoencephalitis, the leading cause of mortality for patients with cryptococcosis (8).

Cryptococcal infections disproportionately affect regions of limited medical infrastructure, especially those with a higher burden of HIV/AIDS (8, 9). Immune deficiency of any kind is a risk factor, but patients with HIV/AIDS primary infections and *Cryptococcus* spp. secondary infections result in nearly 200,000 fatalities annually (8). Additional risk factors for cryptococcosis include organ transplantation, liver disease, renal failure, and treatment with corticosteroids or other immunosuppressive agents. Rates of cryptococcus-related mortality have decreased with the success of anti-retroviral therapies in treating HIV/AIDS, but there remains a notable lack of treatments for *C. neoformans* specifically (10). The most common drugs for treating these infections are expensive and difficult to source in low- and middle-income countries (11). The looming threat of antifungal resistance further highlights the need to better understand cryptococcal infections and develop better therapeutics (12, 13).

Serological evidence suggests that over 50% of children encounter *C. neoformans* by the time they are 5 years old (14). For most, the infectious particles are inhaled and quickly destroyed in the lungs, eliminating any possibility of prolonged infection. For others, the fungi persist in the lungs, hidden from the immune system for years without consequence. Cryptococcal dissemination, when fungal cells escape from the lungs and spread to and colonize extrapulmonary organs, is complex and poorly understood. Because the disseminated, meningitis stage of the infection is both the driver of mortality and the presenting illness (15), a more sophisticated knowledge of this process is necessary to develop new treatment strategies. In the following review, we will discuss the latest research concerning cryptococcal dissemination and highlight unanswered questions on the topic. We will also compare infections with *C. neoformans* to other fungal pathogens in an effort to highlight discoveries that could further cryptococcal research. We divided our discussion into the main steps required for dissemination: (i) entry into and colonization of the lungs, (ii) escape from the lungs and survival in the bloodstream, and (iii) colonization of extrapulmonary organs. Failure at any stage blocks dissemination.

## AT HOME IN THE LUNG

### Pulmonary adaptations

Survival in host physiologic conditions is one of the most important attributes of *C. neoformans* as a persistent fungal pathogen. One key virulence trait is the formation of titan cells, a giant, polyploid cell state that is too large to be phagocytosed by alveolar macrophages. Titan cells are discussed in further detail below in “Setting the stage for dissemination.”

Another critical virulence trait is the ability to grow at mammalian body temperatures. This temperature survival is necessary, but not sufficient, for infection. Many of the critical thermotolerance mechanisms are discussed elsewhere (16, 17). However, several recent studies demonstrated a link between thermotolerance and innate immune evasion. Bloom et al. reported that Ccr4, an mRNA deadenylase, degrades ribosomal protein transcripts in response to host-temperature stress (18). In a mutant *ccr4*∆ *C. neoformans* cell, cell wall β-glucans were exposed following temperature stress (18). β-Glucans are a primary component of the fungal cell wall, and they are normally concealed by the polysaccharide capsule of *C. neoformans* (19). Exposure of these immunogenic molecules increased the uptake of fungi by macrophages expressing the β-glucan receptor dectin-1 (18). Ccr4 also regulates mRNA transcripts necessary for the oxidative stress response. The high osmolarity glycerol (HOG) pathway is similarly involved in both temperature response and cell wall adaptation (20). These data suggest that adaptations to environmental stressors and immune evasion are closely related processes.

*C. neoformans* cells survive and disseminate in anti-inflammatory immune environments. Therefore, evasion and inhibition of pro-inflammatory immune responses are critical to pathogenesis. The *C. neoformans* capsule contributes to both of these functions. Capsule is composed of a layer of polysaccharides called glucuronoxylomannan (GXM) and glucuronoxylomannogalactan (GXMGal), which conceal pathogen-associated molecular patterns (PAMPs) of the cell wall (21). Examples of these PAMPs include the previously mentioned β-glucans, mannoproteins, and chitin. Capsule polysaccharides are branched, and their organization varies depending on the environment and fungal serotype (22). In addition to concealing immunogenic molecules, the capsule promotes persistence by increasing fungal cell size and inhibiting phagocytic uptake. Just as with polysaccharide organization and composition, *C. neoformans* can modulate the thickness of its capsule based on environmental cues (23). Capsule thickness can range from 0 to 30 µm, and cell size varies greatly as a result.

Other pathogenic fungi have evolved similar virulence mechanisms. *Candida albicans* is an obligate commensal fungus that turns opportunistic pathogen when its host is immunocompromised. Rather than rely on a capsule, *C. albicans* conceals β-1,3-glucans with an outer layer of mannan in its cell wall. It also preemptively induces PAMP remodeling after exposure to host-like environmental conditions (24). *C. albicans* makes β-1,3-glucans less detectable by macrophage Dectin-1 through an epitope removal process called “shaving.” The shaving of these epitopes is mediated by the secreted exoglucanase, Xog1, following exposure to lactate or hypoxia (25). Similarly, *Histoplasma capsulatum* conceals β-1,3-glucans through a combination of an outer α-1,3-glucan layer and the expression of a β-glucanase called Eng1 (26). This β-glucanase acts by hydrolyzing β-1,3-glycosyl linkages and enhancing the ability of *H. capsulatum* to evade macrophage detection (26).

### Granulomas

Data indicate that many clinical *C. neoformans* infections are established latently and only manifest under the context of immune suppression (27). Latent *C. neoformans* cells occupy granulomatous lesions in several regions of the lungs, including the alveolar sacs, the interstitium, and the subpleural space between the lungs and the thin layer of tissue that cover them (28, 29). Fungi in these pulmonary “granulomas” can be encased in a complex of macrophages, multinucleated giant cells, neutrophils, and lymphocytes, but are sometimes free from host cells altogether (30). These structures, however, are far from static. They are highly heterogeneous between patients and there is an inverse relationship between fungal containment and granuloma complexity (31). In general, increased host cell trafficking through granulomas decreases the likelihood of fungal replication and disseminated infection.

Granuloma formation is conserved for many dimorphic fungi, including *Coccidioides*, *Blastomyces dermatitidis,* and *H. capsulatum*. In *Coccidioides* infections, both morphotypes, endospores and spherules, are found contained within granulomas (32). Approximately 5% of these infections are thought to involve granuloma formation and their maintenance depends on pro-inflammatory cytokine signaling (33). Similarly, *H. capsulatum* granulomas are populated with pro-inflammatory, IFN-γ-producing CD4^+^ T cells and TNFα-producing macrophages (34). Granuloma structure is also conserved between these infections. Fungal granulomas are often characterized by necrotizing regions, where fungal cells and dead immune cells aggregated at the center are surrounded by infiltrating macrophages and lymphocytes (35).

Granulomas control *C. neoformans* infections, but they can also work against the host by sealing fungi away from clearance mechanisms. Put simply, granulomas are an evolutionary compromise whereby immune cells can contain pathogens and prevent their spread but also protect them from eradication (34). Fungi can survive latently in granulomas for many years (35). In cryptococcal infections, their formation is dependent on granulocyte-macrophage colony-stimulating factor (GM-CSF-signaling), which increases the expression of macrophage receptors specific to the fungal cell wall (36, 37). It remains unclear what other factors control latency and the fate of cryptococcal granulomas, but ongoing research aims to understand these processes through the study of fungal adaptations to their hosts. For more information on cryptococcal granulomas, see “The granuloma in cryptococcal disease” by Ristow and Davis (30).

There are many factors that control cryptococcal survival during infection. PAMP concealment, thermotolerance, and granuloma induction all contribute to the pathogen’s capacity for extended survival in immunocompetent hosts. Immunosuppression then drives reactivation of a latent infection and/or susceptibility to re-infection, providing an opportunity for fungi to proliferate in the lungs and disseminate throughout the host.

## SETTING THE STAGE FOR DISSEMINATION

Escape from the lungs is an essential step in *C. neoformans* pathogenesis. The prevailing theory of cryptococcal dissemination is that fungi escape the lungs into the bloodstream, where they spread throughout the body. How fungi reach the bloodstream appears to be dependent on whether they are yeast or spores. Walsh et al. demonstrated that mice infected intranasally with spores see the accumulation of fungi in lung-draining lymph nodes 7 days post-infection (5). They propose that inhaled spores are trafficked to lymph nodes by alveolar macrophages and, from there, gain access to the bloodstream. There are also limited reports of lymphadenitis as the presenting illness for disseminated cryptococcosis in patients with HIV (38–40). Lymph node invasion is also reported in otherwise healthy pediatric patients, although lymphadenitis in those cases is accompanied by invasion of other organs (41). Overall, it remains difficult to determine how lymph nodes contribute to dissemination in humans.

### Macrophage polarization

Mechanisms by which yeast cells escape the lungs are also poorly understood, but hypotheses include trafficking by host cells or binding to and damaging human lung epithelial cells to allow direct passage into the bloodstream (42–44). Pulmonary escape is typically preceded by some form of immune insult, with T lymphocyte depletion or suppression being the primary risk factor for cryptococcosis (45). The cooperation of T-helper 1 (Th1) cells and macrophages in mounting a pro-inflammatory effector immune response is crucial for disease elimination (46). Pro-inflammatory macrophages (M1) rely on signaling from Th1 cells to initiate and maintain activation through the production of interferon-gamma (IFNγ), tumor necrosis factor-alpha (TNFα), and interleukin 12 (IL-12) (47–49). Under these conditions, phagocytic macrophages traffic to the site of inflammation and produce antimicrobial molecules like nitric oxide (NO) to dispatch pathogens (50). This relationship between T lymphocytes and macrophages, though here simplified, is necessary to clear fungi or contain them in granulomas. When the relationship is disrupted, like in HIV/AIDS, macrophages are not M1 polarized, and they fail to mount a sufficient cell-mediated response against *C. neoformans* (51).

Macrophages exhibit an M2 polarization during cryptococcosis (52, 53) that is driven by *C. neoformans* virulence factors like the secreted proteins, Ssa1 and Cpl1 (54, 55). M2 macrophages are maintained through the cytokines interleukin 4 (IL-4), interleukin 13 (IL-13), and interleukin 33 (IL-33) (49, 56). Rather than NO, M2 macrophages produce the anti-inflammatory molecules arginase 1 (Arg-1) and interleukin 10 (IL-10). These effector molecules suppress lymphocyte activation and the inflammatory response, resulting in uncontrolled fungal growth (57, 58). Fungi are less frequently phagocytosed by M2 macrophages and are more likely to survive when uptake does occur (59).

Several studies have demonstrated that macrophage polarization is dynamic throughout the course of cryptococcal infection (51, 60). Davis et al. showed that macrophages have the capacity to switch between M1 and M2 polarization states as they are exposed to different cytokines (51). This polarization plasticity directly affects microbicidal activity, as NO production varies based on polarization. Furthermore, the polarization phenotype of individual macrophages is reversible, as is their fungicidal capacity. These discoveries explain how changes to the extracellular environment, like a period of immune suppression, can dramatically impact fungal proliferation and escape.

Previously, we discussed the role of capsule as a strategy for *C. neoformans* survival. Capsule conceals PAMPs present in the fungal cell wall and prevents them from being recognized by macrophages. Conversely, fungal recognition and uptake by macrophages have been shown to be an important determinant of disease progression (61–63). These findings present a paradox in our understanding of cryptococcosis, where fungi rely on both concealment for survival and recognition for dissemination. It is also possible that macrophage uptake alters the fungi themselves. Nielson et al. demonstrated that survival in macrophages enhances virulence and that survivor fungi show increased availability of the PAMPs mannose and chitin (64). The authors suggest that *C. neoformans* moves between intracellular and extracellular states depending on the pulmonary immune environment (64). Further research is needed to parse these complex processes.

### Virulence factors

Pulmonary escape is influenced by several virulence factors specific to *C. neoformans*. In the context of an insufficient anti-inflammatory macrophage response, the fungus displays a remarkable capacity for size pleomorphism. *C. neoformans* assumes sizes between 2 and 100 µm in diameter under these conditions (65). The largest of these cells are called “titans,” and their role in disease is particularly well studied. Titan cells act as anchors for cryptococcal infections in that they are too large to be readily destroyed by macrophages, and they produce daughter cells that maintain the fungal population (44, 66). Titan cells are polyploid but somehow generate phenotypically diverse haploid or aneuploid daughter cells through asymmetric DNA division (44, 67). A diverse population of daughter cells increases the likelihood that at least one will be fit enough to survive the host environment and replicate. Titan cells also play a role in dissemination by altering their capsule structure and thickening cell walls (68). These changes impart a heightened resistance to phagocytic killing and environmental stress and are inherited by titan daughters that exhibit similar host resistance (69).

The polysaccharide, GXM, which comprises 90% of the *C. neoformans* capsular material, is another virulence factor that influences pulmonary escape (70). The fungi produce at least two forms of GXM, one that makes up the capsule and another, exo-GXM, which is released extracellularly (47). Data show that exo-GXM synthesis is regulated and that the polysaccharide is not shed passively from the capsular surface but is instead actively released in this alternate form (71, 72). It can be released either as a soluble polysaccharide or in exosomes (73). Both *in vitro* and *in vivo* studies show that exo-GXM increases anti-inflammatory cytokines and decreases the pro-inflammatory cytokines needed for M1 polarization of macrophages (74, 75).

Exo-GXM also acts on immune cells directly. For example, neutrophil migration is impeded by exo-GXM binding to L-selectin, a cell adhesion molecule important for rolling (76). Monocytes treated with exo-GXM have decreased expression of major histocompatibility complex (MHC) proteins, potentially reducing antigen presentation and thus inhibiting the initiation of the adaptive immune response (77). Finally, exo-GXM accumulates in macrophages during infection, and these cells show increased expression of the apoptotic Fas ligand (FasL) (78). Interaction between FasL and Fas receptor (FasR) on the surface of activated T lymphocytes induces apoptosis in FasR-expressing cells. Interestingly, HIV-infected T lymphocytes express more FasR on their surface (79). This could represent a link between the fungal release of exo-GXM, Fas-mediated depletion of T cells, and the absence of M1 polarized macrophages in cryptococcal infections. By this mechanism, *C. neoformans* exo-GXM could further alter the host environment and make it more conducive to fungal proliferation. More broadly, exo-GXM accumulates in the serum, tissue, and cerebrospinal fluid of cryptococcosis patients (80–82). Concentrations of exo-GXM in patient cerebrospinal fluid are detected in the micrograms per milliliter and frequently persist long after the infection is cleared (83).

An immunomodulatory polysaccharide released by a fungus during mammalian infection is not unique to *Cryptococcus* species. *Aspergillus fumigatus* is a fungal pathogen protected by a polysaccharide remarkably similar to *C. neoformans* GXM. This polysaccharide “sheath” is called galactosaminogalactan (GAG) and is composed of α-1,4-galactose, N-acetyl glucosamine (GalNAc), and galactosamine (GalN) (84). It is expressed by *A. fumigatus* hyphae and coats their surface to act as a barrier from the environment (84). Data have shown that extracellular GAG promotes *A. fumigatus* infection and modulates the host immune response by inducing the expression of anti-inflammatory cytokine, IL-1Ra, inhibiting innate immunity (85). This immunomodulation is similar to cryptococcal infections, whereby cytokine profiles are skewed toward nonprotective, anti-inflammatory responses that are conducive for fungal growth. GAG also alters the immune environment by inducing neutrophil apoptosis, a key effector cell in the clearance of *A. fumigatus* from its host. Soluble GAG increases the expression of MHC class I chain-related molecule A (MIC-A) on neutrophils, which promotes interaction with natural killer (NK) cells. The binding of neutrophil MIC-A to NK cell NKG2D results in Fas-dependent apoptosis of the neutrophil via the caspase-8 pathway (86).

The polysaccharide exo-GXM is not the only *C. neoformans* molecule released into the environment that perturbs host immunity. This fungus releases many effector proteins that improve its virulence; we recommend interested readers several key proteomic studies (87–89). Luberto et al. first described antiphagocytic protein 1 (App1), which accumulates in the serum of infected patients (90). Alveolar macrophages treated *in vitro* with recombinant App1 protein exhibit decreased phagocytosis of *C. neoformans*. Furthermore, Δ*app1* mutant fungal cells are readily phagocytosed by alveolar macrophages (90). In a later study, that same group demonstrated that App1 inhibits phagocytosis through the binding of complement receptor 3 (CR3) (91). This is a compelling finding given that CR3 is an important receptor mediating the phagocytosis of microbes like *C. neoformans* that are not easily targeted by the adaptive arm of the immune system. *C. neoformans* relies on the release of another effector protein in infections. Recently, Dang et al. demonstrated the importance of a protein called Cpl1 that causes the M2 polarization of macrophages, an essential feature of disseminated cryptococcal infections (54). They showed that polarization of macrophages via Cpl1 is driven by interactions with Toll-like receptor 4 (TLR4) on the macrophage surface. This outlines a mechanism by which *C. neoformans* preemptively modulates the immune environment to facilitate its own dispersal. Dang et al. hypothesize that Cpl1 activates TLR4 signaling, driving the phosphorylation of STAT3 and inducing the production of Arg-1 (54).

## ESCAPE FROM THE LUNGS

Resisting phagocytic attacks and modulating the immune response are critical preparatory factors in pulmonary escape, but the act itself is worthy of consideration. How does *C. neoformans* leave the airways? Where do the fungi traffic? Many pathogens are trafficked to lymph nodes for antigen processing and presentation; however, clinical cryptococcal infections rarely present in lymph nodes (92). In fact, there are remarkably few reported cases of cryptococcosis with lymph node involvement. Several clinical studies have reported the appearance of fungi in hilar and mediastinal lymph nodes (28, 41), but the infrequency of these reports suggests four possible models: (i) limited sampling of lymph nodes creates a reporting bias, (ii) *C. neoformans* cells exit the lymphatics early in infection and are therefore not present at the time of autopsy or diagnostic biopsy, (iii) that *C. neoformans* does not use the lymphatics as a means of pulmonary escape in humans and instead escape the airways directly into the bloodstream, or (iv) that fungal presence in lymph nodes is limited and transient, constantly seeding the bloodstream and extrapulmonary organs.

### Adherence to lung epithelium

In escaping the lung, *C. neoformans* must traverse both the lung epithelium and the vascular endothelium to reach the bloodstream. Interactions with the former have not been thoroughly described. The first step in fungal–epithelial cell interactions is the act of adherence. Early studies by Merkel and Scofield indicate that both encapsulated and acapsular fungi adhere to epithelial cells (43). Binding of epithelial cells is dependent on the age of the fungal culture and the temperature of the culture media (43, 93). The adherence of encapsulated fungi is mediated by capsular GXM and can be blocked by pretreatment with the GXM-specific antibody, IgG 18B7 (94). Phospholipase B (Plb1) is also implicated in adherence to epithelial cells. Plb1 is an enzyme secreted by *C. neoformans* and a virulence factor necessary for fungal dissemination. ∆*plb1* mutants have reduced adherence to epithelium compared to wild type (WT) or a reconstituted strain, ∆*plb1^rec^* cells (95). The adherence of acapsular fungi is mediated by mannoprotein 84 (MP84) (96). It remains unclear how *C. neoformans* passes through lung epithelium and whether it depends on epithelial internalization, damage, macrophage uptake, or another mechanism entirely.

### Crossing the vascular endothelium

Up to this point, many of the studies concerning endothelial crossing have focused on exit from rather than entry to the vasculature. Furthermore, the referenced studies focus on endothelial cells of the CNS and not the lungs. Despite differences between these types of endothelium, we think there is value in considering whether these mechanisms apply to bloodstream entry. With that in mind, here are three proposed mechanisms of *C. neoformans* crossing the vascular endothelium into the bloodstream: Trojan horse (intracellular), transcellular, and paracellular migration.

### Trojan horse migration

The proposed mechanism of Trojan horse crossing posits that fungi are phagocytosed in the airways, then carried by the host cell into the blood and extrapulmonary organs (97, 98). The ability of *C. neoformans* to thrive as a facultative intracellular pathogen is essential for the Trojan horse hypothesis. *C. neoformans* cells can survive the acidification and oxidative/nitrosative stress of phagolysosomes (99–101). *C. neoformans* cells are resistant to low pH environments, possibly an evolutionary trait that arose through its association with environmental niches like bird guano (102, 103). The pathogen also appears to disrupt the phagolysosome through passive damage. Davis et al. demonstrated with ratiometric fluorescence microscopy that *C. neoformans* can damage the phagolysosome and that this damage is correlated with fungal replication from inside the compartment (104). They also showed that damage is avoided if the macrophage is supplemented with M1-polarizing IFNγ (104).

Trojan horse migration was first proposed after observation of fungi in host phagocytes (105). *In vivo* studies followed, with an infection model using adoptive transfer of fungi-loaded mononuclear phagocytes (42). These studies determined that loaded phagocytes effectively disseminate fungi (61) and that intracellular dissemination to the lungs, spleen, and brain is more efficient than infection with free yeasts (98). Using an *in vitro* model of barrier crossing, Santiago-Tirago et al. showed definitively that fungi-loaded mononuclear phagocytes can migrate across a barrier composed of the microvascular endothelial cell line, hCMEC/D3, and that this process is enhanced with the addition of TNFα (106). It is possible that this mechanism of migration is also applicable to lung escape and crossing lung epithelium. Although these results are promising, other putative escape mechanisms do not involve the help of host phagocytes.

In order to disseminate via a Trojan horse mechanism, *C*. neoformans cells would also have to escape from their “horse” without incurring damage to themselves. Indeed, they do so via nonlytic exocytosis, termed “vomocytosis.” This process was first described in *C. neoformans* and several closely related species but has since been reported in infections with *Candida krusei* and *C. albicans* (107, 108). Vomocytosis is an immunologically covert means of escape from host cells, as exit occurs without damage or the induction of an inflammatory response. The *C. neoformans* capsule plays a role in this process, as acapsular strains have lower rates of vomocytosis (109). Plb1 is likely important because ∆*plb1* mutants have lower rates of vomocytosis (110). Artificially controlling phagosome pH has demonstrated that basic phagosomal pH results in increased vomocytosis (111). Thus, phagosomal pH also appears to be important for vomocytosis, as it occurs exclusively in non-acidified phagosomes. It was initially described *in vitro* using murine macrophage cell lines but has since been studied in primary cells from mice, humans, birds, and fish (111–115). The zebrafish larvae model has been particularly useful for these studies, as it allows for the imaging of live specimens infected with *C. neoformans*. In zebrafish, Bojarczuk et al. demonstrated that vomocytosis occurs *in vivo*. They measured incidence over 12 hours and found that 5%–15% of fungi-loaded macrophages underwent vomocytosis in that time period (113). Despite its intrinsic connection to intracellular migration, vomocytosis and dissemination appear to be inversely correlated. Gilbert et al. showed that pharmacological enhancement of vomocytosis results in reduced dissemination (115). More work is required to better understand the connection between intracellular migration and vomocytosis and their contribution to cryptococcal dissemination.

### Transcellular migration

*C. neoformans* can also cross vascular endothelium extracellularly. One proposed mechanism of extracellular escape is transcellular migration, by which fungi pass directly through endothelial cells. Here, transcytosis is dependent on the adherence and uptake of fungi by endothelial cells (116, 117). This is mediated by hyaluronic acid (HA), another polysaccharide found in the capsule structure of *C. neoformans* (118, 119). HA is bound by the receptors CD44 and RHAMM on endothelial cells, and binding is followed by actin-dependent internalization of the fungi (120). Most of the research concerning this process has focused on the interaction between *C. neoformans* and brain microvascular endothelial cells. These receptors are expressed by endothelial cells throughout the body, suggesting transcytosis could be a mechanism of escape from the lungs and entry into extrapulmonary organs. Transcytosis of *C. neoformans* can also occur through binding of the extracellular fungalysin metalloprotease, Mpr1 (121). This protein was shown to control attachment and internalization of the fungi by endothelial cells both *in vitro* and *in vivo*. Mpr1 engages host endothelial cell annexin A2 (AnxA2), also promoting transcytosis through F-actin cytoskeleton remodeling (117).

### Paracellular migration

The second extracellular mechanism of vascular endothelium crossing is paracellular migration, which involves the passage of free fungi between endothelial cells. This mechanism requires either active or passive manipulation of the endothelial barrier by *C. neoformans*. In paracellular migration, the junctions between endothelial cells need to be sufficiently damaged to allow passage. An example of passive manipulation of junctions is the observation that *C. neoformans* induces the production of IL-33 from lung epithelial cells (122). IL-33 is a Th2 cytokine that promotes M2 macrophage polarization and an anti-inflammatory immune response. In this case, the presence of IL-33 reduces tight junction E-cadherins, weakening the connections between cells and barrier integrity overall (122). *C. neoformans* exhibits two methods of active barrier manipulation. It produces an enzymatic virulence factor called urease that catalyzes the conversion of urea into carbon dioxide and ammonia (123, 124). Urease helps sequester fungi in microcapillaries, which could facilitate barrier crossing (125). Singh et al. reported that urease compromises the integrity of endothelial barriers by reducing the expression of host tight junction protein, ZO1 (126). The precise mechanism controlling this expression has yet to be defined; however, ammonia derived from urease catalysis is thought to be involved. *C. neoformans* serine proteases also actively manipulate barrier integrity. Rodrigues et al. demonstrated that fungal serine proteases cleave components of the basement membrane and extracellular matrix, including fibronectin, laminin, and type IV collagen (127). Damage to these components increases barrier permeability during cryptococcal infection (128).

Fungal pathogens introduced through inhalation have a shared trait. Dissemination from the lungs is rare but becomes more frequent if the host is immunocompromised. This is true of *C. neoformans* as well as *H. capsulatum* (129, 130), *A. fumigatus* (131, 132), *Coccidioides* species (133, 134), *and B. dermatitidis* (135, 136). It prompts an important question. What about these pathogens makes them so adept at escaping the lungs in this context? As previously outlined, there are several proposed mechanisms of *C. neoformans* migration from the alveolar spaces of the lungs to the bloodstream. The relative contribution of these mechanisms remains poorly understood. We do not know the rate of dissemination from the lungs or whether dissemination is dependent on size diversification.

Pulmonary escape is an important step in cryptococcal pathogenesis. Above, we have reviewed the immune environment necessary for pulmonary escape, virulence factors that modulate the environment, and several potential mechanisms employed by *C. neoformans* to enter the bloodstream. It is clear there is no one virulence factor or escape mechanism that controls cryptococcal dissemination. This complex process requires further research to better understand how an innocuous environmental organism can disseminate dangerously to the bloodstream.

## BLOODSTREAM DISSEMINATION TO EXTRAPULMONARY ORGANS

### Blood

Fungemia, the presence of fungi in the blood, is poorly understood in cryptococcal pathogenesis. Clinical studies reveal that fungal burden in the blood is highly variable among patients. In a cohort of 63 AIDS patients with *C. neoformans* secondary infection, 57% had a fungal burden of <1 CFU/mL of blood (137). In a study of 140 HIV/AIDS patients with secondary infections, *C. neoformans* was isolated from the blood in 20.8% of patients and was the most commonly isolated pathogen (138). Early work on murine models of cryptococcal fungemia determined that fungi can be detected in the blood well after intravenous (IV) inoculation, but that blood fungal burden does not correlate with the inoculating dose just 24 hours post-inoculation (139). This suggests that fungi are quickly cleared from the blood or traverse the vascular endothelium into organs (132). Differential centrifugation and plating of blood from infected mice demonstrated that fungi are both free and associated with leukocytes in the blood (139). In contrast, a multi-year surveillance study of human invasive fungal infections showed that *C. neoformans* was rarely found in blood (3.5% of reported bloodstream infections) but commonly found in cerebral spinal fluid (CSF) (91.7% of reported yeast species isolated from CSF) (140). This discrepancy could be influenced by the location of the study and the time of infection the samples were collected or by differences in the CFU burden used to define fungemia, which was quite sensitive in Lortholary et al. (139). These early studies of cryptococcal fungemia prompt further questions about the state of fungi in the blood. Recently, Francis et al. found free *C. neoformans* cells in the bloodstream at 3 and 7 days post-intranasal inoculation in a mouse infection model (141). Nielson et al. observed that the majority of yeast cells in the bloodstream in a zebrafish infection model are extracellular (142). Both groups, however, found that fungi are quickly phagocytosed by microglia, which might occur just before or just after blood brain barrier crossing. Either way, these data suggest that *C. neoformans* is predominantly not associated with host cells up until barrier crossing and extrapulmonary organ entry.

The penultimate step in fungal dissemination is the seeding of extrapulmonary organs from the bloodstream. Like pulmonary escape, organ seeding requires traversing vascular endothelium. The mechanisms explored above are all accepted as plausible modes of entry to the extrapulmonary organs. Most of the research exploring organ seeding revolves around the CNS, considering *C. neoformans* tropism for that compartment and the fact that meningoencephalitis is both the driver of mortality in cryptococcosis patients and the presenting illness (8, 143). Some discoveries have been made with regard to other organs, and it is important to highlight that work as we explore seeding and the capacity of extrapulmonary organs to act as reservoirs for the pathogen.

### Potential *C. neoformans* reservoirs: liver and others

In the murine inhalation model of cryptococcosis, the liver is one of the first organs seeded after pulmonary escape (144). There is also a correlation between liver diseases and susceptibility to *C. neoformans* infection (145, 146). In rare cases, a liver transplant has transmitted cryptococcosis to the organ recipient despite a lack of active disease in the donor (147), suggesting the possibility that *C. neoformans* cells can lie latent in the liver. As a surveillance organ for intravascular infections, the liver filters microbial pathogens from the blood. Recently, Sun et al. confirmed that this filtration extends to fungi like *C. neoformans* (148). Using intravital microscopy, they demonstrated that disseminating fungi are trapped in the liver sinusoids and later phagocytosed by Kupffer cells. This finding provides an explanation for how the liver could become a reservoir for fungi in cryptococcal infections. If Kupffer cells are ineffective at killing phagocytosed fungi, they could escape via a mechanism like vomocytosis and proliferate in the liver parenchyma. Despite their shared resident macrophage lineage, Sun et al. also showed that Kupffer cells do not rely upon IFNγ for clearance of *C. neoformans* (148). This is an important observation because it suggests that Kupffer cells may be less susceptible to T cell deficiency-driven M2 polarization than alveolar macrophages. IFNγ is a critical T cell-derived cytokine for alveolar macrophages and their clearance of fungi from the lungs. Both the lungs and the liver rely on resident macrophage populations for defense, but this difference in cytokine reliance could be exploited with future therapeutics aimed at augmenting Kupffer cell function.

In 2022, our laboratory defined a subset of *C. neoformans* that arises during infection (149). These “seed cells” exhibit an enhanced ability to enter and colonize extrapulmonary organs. They are characterized by their small size (~7 μm) and increased surface mannose exposure. Subsequently, we demonstrated that liver-resident macrophages phagocytose seed cells at a higher rate than other *C. neoformans* cell populations, possibly as an attempt by the immune system to sequester invading fungi. However, the increase in phagocytosis is accompanied by robust seed cell growth in the liver, suggesting that fungal containment ultimately backfires for the host.

The prostate has also been implicated as a *C. neoformans* infection site. Although relatively rare, there are several clinical case reports highlighting cryptococcal prostatitis in immunocompromised patients (150–155). These cases are characterized by granulomatous inflammation of the prostate tissue and foci of necrosis. If fungi are sequestered in granulomas in the prostate, it could be a refuge from antifungal treatment and a site of relapse (152). Although these studies do not conclusively establish the liver or prostate as reservoirs for *C. neoformans*, they do suggest further study is warranted.

### Spleen

Spleens are secondary immune organs that are also seeded by *C. neoformans*. Similar to the liver, the spleen is responsible for filtering blood, but it is also rich with macrophages and lymphocytes that work in tandem to mount adaptive immune responses (156). Because of these dual functions, spleens are likely important in responding to early disseminated cryptococcal infections. Generally, spleens are composed of a red pulp region that mechanically filters blood, a white pulp region of lymphocytes, and a marginal zone of macrophages that divide the two (156–158). Red pulp macrophages take up GXM after intravenous inoculation with the polysaccharide (158). Based on the previously mentioned immunomodulatory properties of GXM, this suggests that red pulp macrophages are rendered ineffective in disseminated cryptococcosis. Without these macrophages, fungi likely traffic the bloodstream and seed extrapulmonary organs more easily. Although there is currently no data suggesting splenic colonization, understanding how cryptococcosis inhibits the immune cells in this space could be relevant to dissemination.

### Central nervous system

Meningoencephalitis is the main driver of mortality in cryptococcal infections. This clinical outcome begins with fungal traverse from the bloodstream, across vascular endothelium, and into the brain or spinal cord. The endothelial barrier protecting the CNS is called the blood-brain barrier (BBB) and is composed of vascular endothelial cells, pericytes, and glial cells (159). In disseminated cryptococcosis, fungi can be found in the brain microvasculature, parenchyma, abluminal space, and the CSF. *C. neoformans* proliferates rapidly in the CNS, in part because of its ability to metabolize inositol, a six-carbon sugar that is abundant in the central nervous system (160–162). Another factor contributing to fungal proliferation is that the CNS does not mount strong immune responses like the rest of the body. Circulating mononuclear cells (i.e., lymphocytes, neutrophils) can enter some parts of the CNS, but the brain parenchyma is largely free of immune cells other than tissue-resident microglia (163).

Given that microglia are protective against various viral, bacterial, and fungal infections, it is expected that they are similarly protective against *C. neoformans*. A publication by Mohamed et al. suggests otherwise (164). The authors explored the role of microglia in cryptococcosis and found that they did not have a protective effect against cryptococcal meningitis. Instead, depletion of microglia results in a significant decrease in brain fungal burden (164). They subsequently showed that this decrease in fungal burden is dependent on a lack of intracellular growth within microglia (164). Without microglia, *C. neoformans* lacks an intracellular niche where it is protected from copper starvation and other stressors. These observations likely explain why *C. neoformans* grows relatively unchecked once it crosses the BBB into the brain parenchyma.

Gibson et al. recently described a mechanism of CNS seeding that relies on blood vessel occlusion and mechanical disruption of the BBB (165). Their work suggests that disseminating fungi become trapped in the vasculature and proliferate, resulting in vasodilation and stress damage to the endothelium. They also propose that hemorrhagic dissemination into the brain parenchyma is part of a positive feedback loop in which vasodilation following infection results in increased tension of the endothelium and more frequent seeding of the CNS (165). Such damage is abated if the immune system can intervene before the BBB is disrupted. An example of this intervention was described in a study by Zhang et al., where neutrophils were shown to clear fungi from brain microvasculature (166). More specifically, neutrophils recognized complement C3-opsonized *C. neoformans* attached to the luminal wall, engulfed the fungi, and then returned to the circulation (166). This study also showed that neutrophil depletion enhanced brain fungal burden, highlighting their importance to fungal clearance.

In contrast, Nielson et al. recently demonstrated the importance of phagocytes in seeding the CNS of zebrafish (142). They observed an infected monocyte/macrophage extend a pseudopod from the vasculature into the brain parenchyma and carry fungi across the BBB. Similarly, they observed parenchymal microglia traffic to areas where fungi were bound to the endothelium and internalize them. From this work, they surmise that CNS seeding involves several complementary mechanisms rather than one definitive mechanism. In their model, fungi must first travel through the bloodstream and bind to endothelial cells in the brain microvasculature. The act of binding is followed by BBB crossing via transcellular or paracellular migration. Nielson et al. also showed that the Trojan horse is actually two distinct phagocyte-specific mechanisms involving either circulating monocytes/macrophages or resident microglia (142). Francis et al. further defined a role for phagocytes as the primary immune cells responding to CNS seeding (141). These researchers utilized tissue clarification and decolorization techniques to enable unprecedented high-resolution imaging of murine tissue infected with *C. neoformans* (141). They observed that a majority of fungi, infected intravenously, can reach the brain and cross the BBB within 24 hours. Furthermore, these newly seeded fungi are associated with brain-resident ionized calcium-binding adapter molecule 1 (Iba1^+^) macrophages (141). Whether these Iba1^+^ macrophages are microglia or a closely related brain border-associated macrophage subset remains unclear. These findings are valuable contributions to our understanding of organ seeding in cryptococcosis.

## CONCLUSIONS

*C. neoformans* pathogenesis relies on a diverse set of virulence factors to escape the lungs, disseminate through the bloodstream, and seed extrapulmonary organs. How host immune systems respond to these virulence factors and change the course of infection further complicates our understanding of the disease. Some major outstanding questions in the field include (i) the relative contribution of the Trojan horse versus free fungal cell crossing of barriers from the lungs into the bloodstream and from the bloodstream into extrapulmonary organs, (ii) the extent to which fungal cells disseminate via the lymphatic system versus the bloodstream, and (iii) the role of fungal cell attributes, such as morphology, in the dissemination process. Moreover, the severe CD4^+^ T cell deficiencies experienced by the vast majority of cryptococcal meningitis patients complicate current attempts to evaluate interactions between the host immune system and fungal cells, as the role of the adaptive immune system could well be extremely limited. Further research is needed to understand and ultimately block the spread of fungal cells during cryptococcosis.
